# Numerical Analysis of the Bond Strength between Two Methacrylic Polymers by Surface Modification

**DOI:** 10.3390/ma14143927

**Published:** 2021-07-14

**Authors:** Joanna Taczała, Katarzyna Rak, Jacek Sawicki, Michał Krasowski

**Affiliations:** 1 Institute of Materials Science and Engineering, Lodz University of Technology, Stefanowskiego 1/15, 90-924 Lodz, Poland; joanna.taczala@dokt.p.lodz.pl; 2 Faculty of Medicine Division of Dentistry, Medical University of Lodz, Tadeusz Kościuszko Ave., 90-419 Lodz, Poland; katarzyna.rak@onet.eu; 3 University Laboratory of Material Research, Medical University of Lodz, Pomorska 251, 92-216 Lodz, Poland; michal.krasowski@umed.lodz.pl

**Keywords:** acrylic dentures, numerical analysis, FEM, surface development, shear test

## Abstract

The creation of acrylic dentures involves many stages. One of them is to prepare the surfaces of artificial teeth for connection with the denture plates. The teeth could be rubbed with a chemical reagent, the surface could be developed, or retention hooks could be created. Preparation of the surface is used to improve the bond between the teeth and the plate. Choosing the right combination affects the length of denture use. This work focuses on a numerical analysis of grooving. The purpose of this article is to select the shape and size of the grooves that would most affect the quality of the bond strength. Two types of grooves in different dimensional configurations were analyzed. The variables were groove depth and width, and the distance between the grooves. Finally, 24 configurations were obtained. Models were analyzed in terms of their angular position to the loading force. Finite element method (FEM) analysis was performed on the 3D geometry created, which consisted of two polymer bodies under the shear process. The smallest values of the stresses and strains were characterized by a sample with parallel grooves with the grooving dimensions width 0.20 mm, thickness 0.10 mm, and distance between the grooves 5.00 mm, placed at an angle of 90°. The best dimensions from the parallel (III) and cross (#) grooves were compared experimentally. Specimens with grooving III were not damaged in the shear test. The research shows that the shape of the groove affects the distribution of stresses and strains. Combining the selected method with an adequately selected chemical reagent can significantly increase the strength of the connection.

## 1. Introduction

Patients who come into a dental practice have several denture options to choose from. The simplest, most popular and cheapest [1] form of treatment is an acrylic denture, consisting of acrylic fake teeth and an acrylic denture plate. A more expensive but superior form of prosthetic reconstruction is a removable denture metal framework. These dentures are made of a metal framework designed by a dental technician, an acrylic denture plate and acrylic artificial teeth [2]. This paper focuses on prosthetic reconstruction using acrylic dentures because the problem of connecting two similar materials occurs in this type of treatment [3,4,5].

The process of creating acrylic dentures involves several stages [6,7,8]. One of them is the preparation of the surfaces of artificial teeth. This stage is very important, as the strength of the bond between the teeth and the denture plate depends on the correct preparation of the surfaces. Dentures are expected to last for 4 to 5 years [9,10], as after this period they cause a loss of height of the alveolar process and/or the grinding down of abutment teeth in the case of partial dentures. However, some patients report that their dentures require repair after only a few months, with 33% of these cases caused by a tooth falling out [11,12].

Given the above issue, numerous researchers have attempted to analyze the process of bonding acrylic teeth to the denture plate to devise an optimal way to increase the strength of the bond between these materials. Methods proposed by these researchers may be divided into two groups: chemical and mechanical.

Chemical methods involve rubbing the surface of the tooth with a suitable chemical substance. These most commonly used include:

- methyl methacrylate (MMA) [13,14],
- ethylene glycol dimethacrylate (EGDMA) [10],
- a monomer (i.e., a combination of MMA and EGDMA) [12,15,16,17,18],
- acetone [13,19,20], or
- glues [13,19,20] supplied by various manufacturers of acryl (composed primarily of MMA).

MMA had the most significant effect on increasing the strength of the bond. For example, in a study comparing the resistance of substances to denaturation, the strength of samples treated with MMA increased to 14.47 N. In contrast, the strength of samples treated with other substances ranged from 8.51 to 11.42 N (strength of untreated samples amounted to 8.79 N) [13].

The chemical method also had the best results when compared with other ways of improving the strength of the bond (control = 30.06 MPa, sandblasting = 33.52 MPa, methylmethacrylate based adhesive agent = 42.44 MPa, surface treated with an Er,Cr:YSGG laser system = 35.71 MPa) [21]. The use of chemical substances aims to activate existing polymer bonds, create new chains, and treat the surface of acryl [22]. MMA and EGDMA are the most popular substances, as acryl is mainly composed of polymethyl methacrylate (PMMA).

Fibers that imitate gums or various types of filler (e.g., cellulose [23]) are also sometimes used. When creating the denture plate, the technician combines MMA with a cross-linker in the form of EGDMA. When subjected to a high temperature and pressure, the substance undergoes polymerization, creating the final product [24]. Therefore, treating the surface may contribute to increasing the grip between the two materials [25,26].

Some researchers suggest combining chemical methods with the second group, i.e., so-called mechanical methods [27].

Mechanical methods include two additional types of treating the surface of teeth: creating a retaining groove and surface development. Retaining grooves are formed by drilling a groove in a tooth using a burr on the side adjoining the plate. They are created to create a pocket into which acryl will flow. Grooves shapes may be round, V-shaped, T-shaped, or shaped as a diatoric cavity. However, studies show that the shape of the groove has no impact on the strength of the bond [5].

The final method of improving the strength of the bond discussed in the paper is mechanical surface development. Studies show that appropriate development of the surface significantly increases the strength of the bond, including vonds between dissimilar materials [28]. When bonding acryl with acryl, researchers have identified the following methods:

- shearing the enamel layer using a burr or sandpaper [12,13,18,27,29],
- sanding using aluminium oxide [12,13,18,21,29,30,31].

Sanding of the surface may significantly increase the surface development, thus contributing to an improved bond between the two materials [32,33]. However, in this process, aluminium oxide particles are driven into the surface of the treated material [34], which in the case of acryl may reduce the surface are directly adhering to PMMA. Ultimately, such treatment may have no effect on the bond between two acrylic surfaces or it may even even reduce its strength.

There are also problems with standardizing the results of these tests, as researchers use various methods to test the strength of the bond. They also applied to sand using differing gradations at differing angles and in many cases, the papers do not contain any information about the burrs and sandpaper used to shear the enamel layer.

Of note is the fact that some researchers used an Er,Cr:YSGG [21] or Er:YAG [13] laser in their methods. Unfortunately, this method is very expensive, and lasers of this type are not used in dental laboratories. Due to this, the method cannot be successfully adopted in practice even if it produces satisfactory results.

Grooving is a method that has so far not been used in dental technology despite producing interesting results. Thin grooves can be created using small discs available in every Dental Technician’s workshop. The best and most economical method that enables the analysis and optimization of multiple variants of such surface modification is the application of computer software that uses the finite elements method (FEM) [35,36].

Therefore, this paper aims to apply FEM to analyze surfaces modified using the mechanical grooving method. The analysis will produce optimal grooving parameters that will improve the strength, quality, and functionality of medical devices.

## 2. Materials and Methods

**Figure 1:**
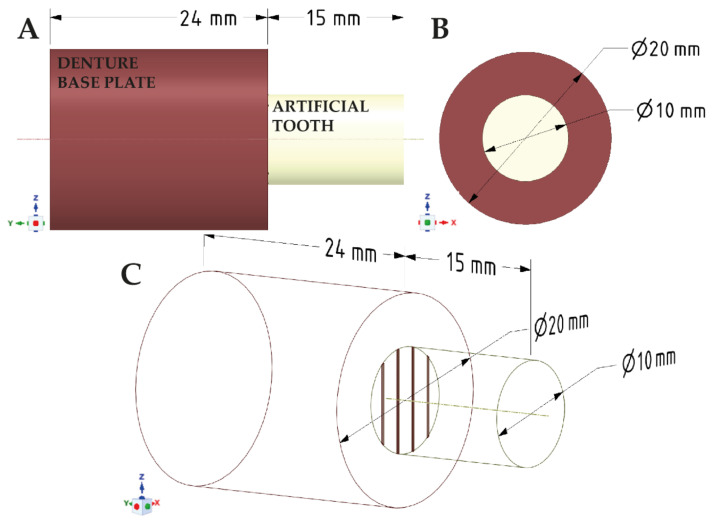
Geometric model of samples used for numerical analysis. Elements representing the denture base plate and the artificial tooth in the (**A**) X-axis, (**B**) Y-axis and (**C**) a sketch.

A 3D model of two polymer samples with various mechanical modifications, depicting a bond between the treated surfaces, was created together with a discrete model using ANSYS Workbench software with the SpaceClaim module (Ansys Inc., Canonsburg, PA, USA). The model consisted of two solid figures used to represent the shear strength test—Figure 1.

**Figure 2:**
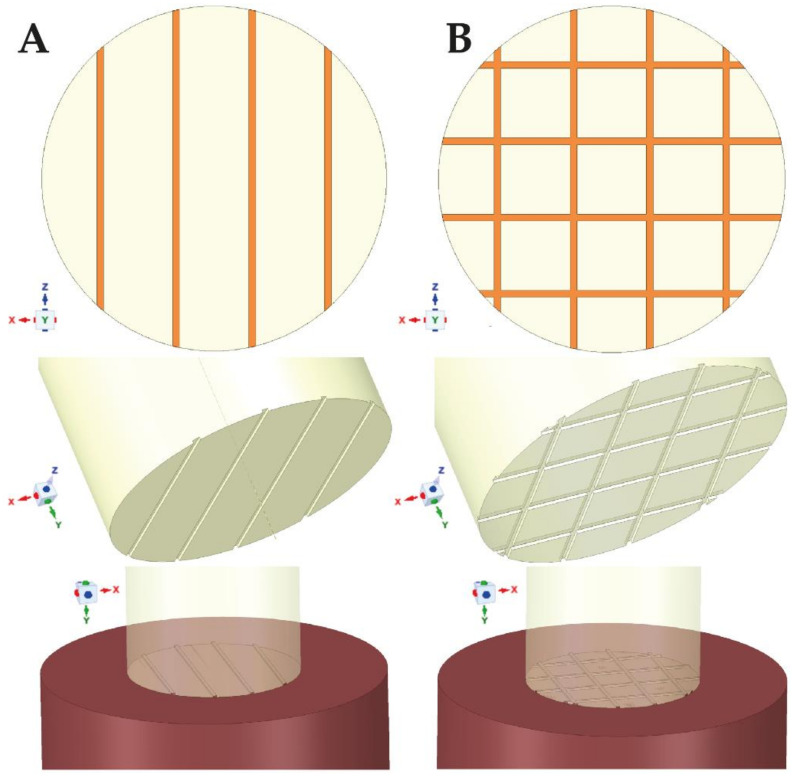
Examples of (**A**) parallel (marked as ‘III’), (**B**) cross grooving (marked as ‘#’).

Two types of grooves (parallel—marked with ‘III’, and cross—marked with ‘#’) (Figure 2) were analyzed in various dimensions, using the variable parameters of the depth and width of the grooves and the distance between the grooves. The grooving variants subjected to analysis are specified in Table 1.

**Figure 3:**
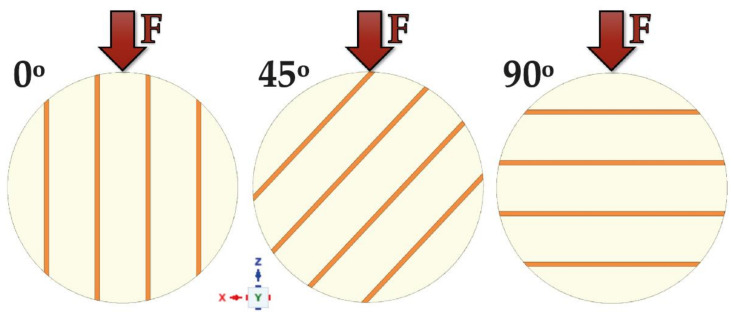
Force point variants.

Furthermore, each model was analyzed in terms of its angular position regarding the force load affecting it. Three angular variants of force load were tested: along the *Z*-axis (0°), displaced by 45° along the ZX plane, and displaced by 90° along the *Z*-axis, i.e., along the *X*-axis only in respect of parallel grooving (Figure 3).

**Figure 4:**
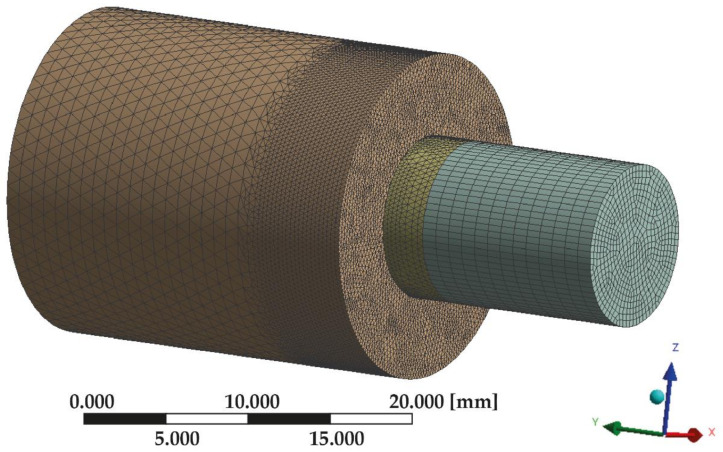
Discrete model.

The geometric model was then reformatted into a discrete model. The generated grid consisted of adequately parameterized finite elements, enabling favorable and precise simulation results. Additionally, with the MultiZone function, mesh subzones were defined which, combined with the earlier division of the geometry, enabled the optimization of the grid. The most significant area of accuracy and precision of the grid components was the location where the two cylinders met, i.e., the grooved surface. The mesh was denser, and the dimensions of elements were reduced in these locations (Figure 4). Mesh convergence analysis was carried out by incrementally increasing the number of elements and verifying the estimations to ensure the convergence of the numerical solution. Finally, mesh convergence tests were performed, resulting in a total number of elements and nodes of 448,957 and 300,985, respectively.

**Figure 5:**
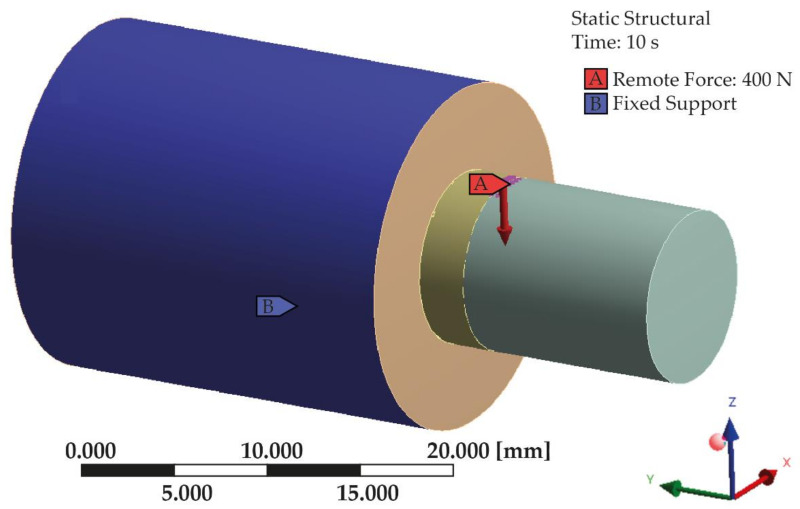
Boundary conditions and loads of the analyzed model.

The model was restrained on the large cylinder walls along the circumference using the ‘fixed support’ option. The load was applied using the ‘remote force’ option. A force of 400 N was applied to the model along the *Z*-axis to the outer surface of the small cylinder, as shown in Figure 5. The force was based on the conditions found in the oral cavity [3,5,10]. A “bonded” contact was used at the junction of both bodies.

The material data of both components matched the data of the prosthetic acryl used in the heat-curing method. The acryl material was modeled as an isotropic material with a bilinear elastic-plastic stress-strain curve with the strain hardening constituents of the viscoplastic model. Detailed parameters of the acryl used in the model, based on papers [3,5], are shown in Table 2.

Numerical analyses were performed to determine the distribution of stresses (equivalent von Mises) on the bond between the large and small components and the values and distribution of deformations.

The best configuration obtained from the numerical simulation giving the best results and the exact configuration of grooves with second types of grooves were also tested with a universal testing machine (from Zwick/Roell).

The shear test was carried out according to the rules for composite materials. The machine knife displacement rate was set to 2 mm/min (the ISO standard for the connection of artificial teeth with the denture base plate (ISO 22112:2017) requires the use of a displacement in the range from 0.5 to 10 mm/min). According to the manufacturer’s leaflet, the samples were made of the Vertex Rapid Simplified heat cure acrylic resin (Vetex Dental, Soesterberg, The Netherlands).

**Figure 6:**
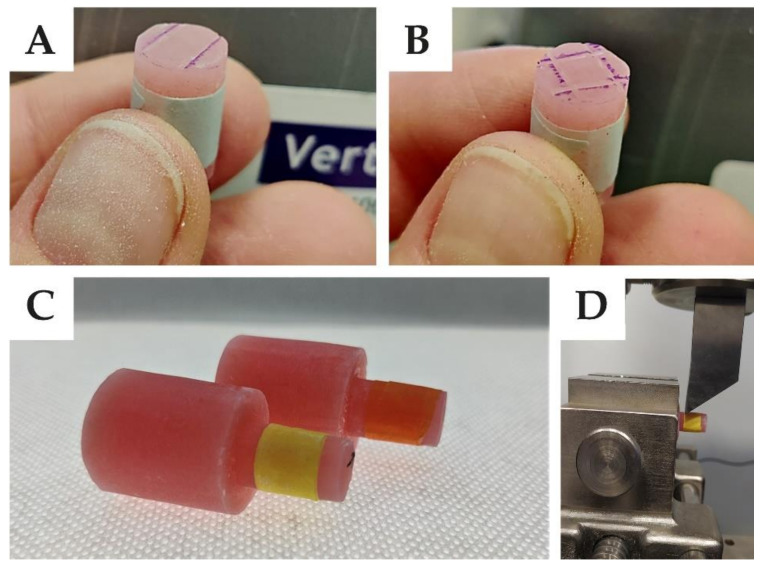
Created groves: (**A**) III according to no. 2, (**B**) # according to no. 14. (**C**) final samples, (**D**) shear test.

The samples (Figure 6) were mapping as per the simulation (dimensions as in Figure 1). On the surface of the smaller cylinder, before pouring acrylic from the more significant part, grooves were made with a rotating disc (the thickness of the disc was 0.2 mm). The grooves were made according to the dimensions of the variants no. 2 from the III group (width 0.20 mm, distance 5.00 mm, depth 0.10 mm) and no. 14 from # group (width 0.20 mm, distance 5.00 mm, depth 0.10 mm). For the III-group sample, the force was applied at 90°, while force was applied to the #-group sample at 0°.

## 3. Results

**Figure 7:**
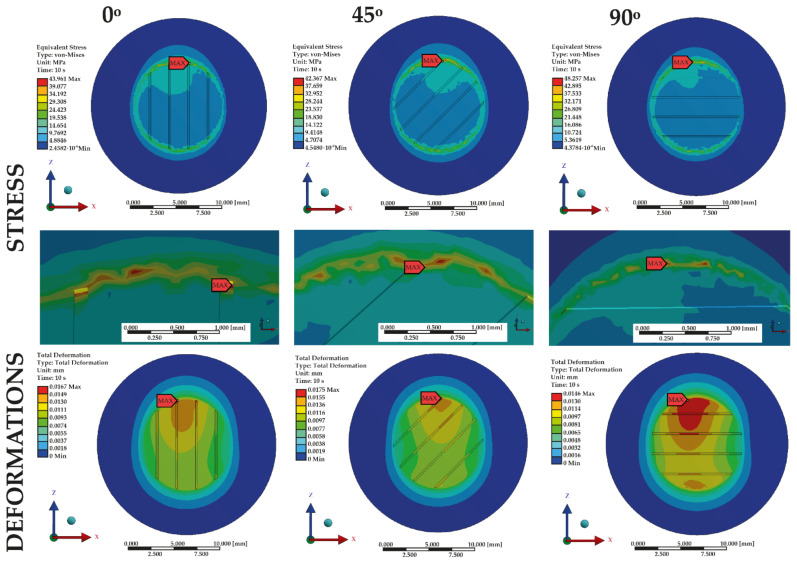
Distribution of stresses (whole view and focus on area of maximum stress) and deformations depending on the direction of the applied force, based on grooving variant 1.

The observation was focused on the highest stresses present on the surface with the protruding grooves and maximum deformations to which the material was subjected. The results presented are shown on the surface of the models corresponding to the base plate, because the highest stresses and deformations appeared in this area (at the connection of the tooth and the denture base plate). All views are from the Y-axis perspective. Results for each sample were then compared, depending on the angular position. The generated maps show a distribution of stresses typical of shear strength tests. The test results help locate the points where the highest stresses appear, causing excessive loads to be applied to the material and, as a result, potentially damaging the sample. Sample maps of the impact of the angle of application of the force generated based on variant no. 1 (parallel grooving) are shown in Figure 7.

**Figure 8:**
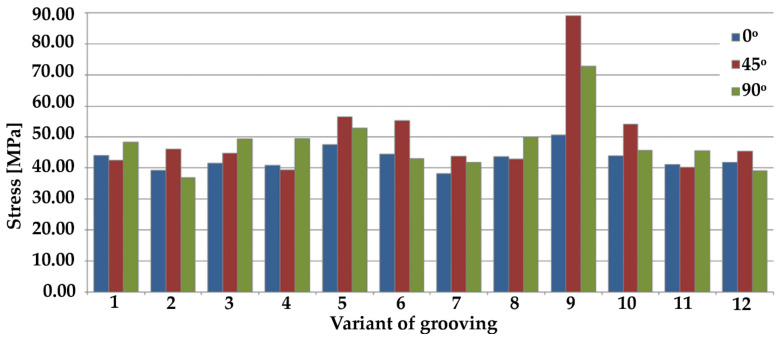
Overview of maximum stresses from simulation results of parallel grooving (III) at various angles.

**Figure 9:**
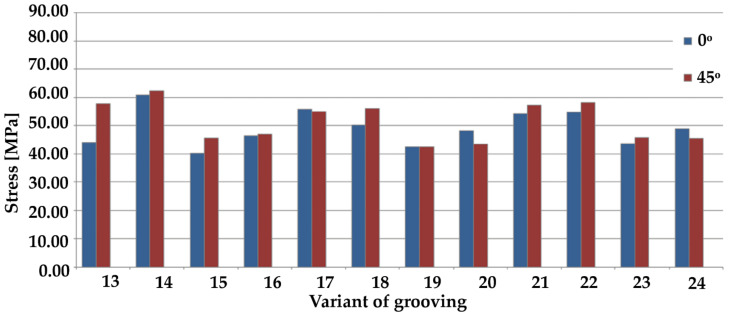
Overview of maximum stresses from simulation results of cross grooving (#) at various angles.

**Figure 10:**
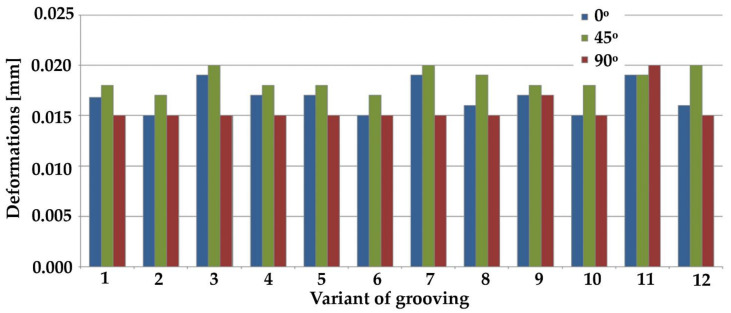
Overview of maximum deformations from simulation results of parallel grooving (III) at various angles.

**Figure 11:**
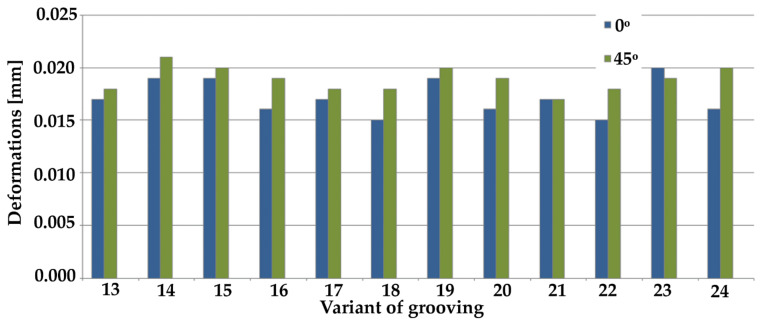
Overview of maximum deformations from simulation results of cross grooving (#) at various angles.

Figure 8 and Figure 9 show maximum stresses, while Figure 10 and Figure 11 show maximum deformations.

Considering only the samples with grooves positioned at an angle of 0°, sample 7 recorded the best result with the lowest maximum stress, whereas sample 14 recorded the worst result by far. Regarding tests on samples with grooves positioned at an angle of 45°, the highest stresses were generated on sample 9, while the lowest were on sample 4. A comparison of samples with grooves positioned at an angle of 90° indicates that the lowest maximum stresses were present on sample 2, whereas the highest were present on sample 9. This was also the result in the case of the grooves positioned at an angle of 0°.

When comparing the results from all angles, the lowest stresses were produced when using the following variants:

- no. 2, with grooves positioned at an angle of 90°,
- no. 4, with grooves positioned at an angle of 45°,
- no. 7 and no. 15, with grooves positioned at an angle of 0°.

The results of the tests for maximum deformation based on grooving input positioning at an angle of 0° indicate similar values of minimum deformations in the case of samples 2, 6, 10, 18, and 22. The highest value in respect of this angle was observed in the case of variant 23. Regarding the deformations in the 45° samples, the highest deformation value was recorded for sample no. 14, whereas the lowest values were recorded for samples 2, 6, and 21. With regards to the 90° samples, the results were in most cases very similar for all tested samples. The highest deformation value was recorded in sample 11.

**Figure 12:**
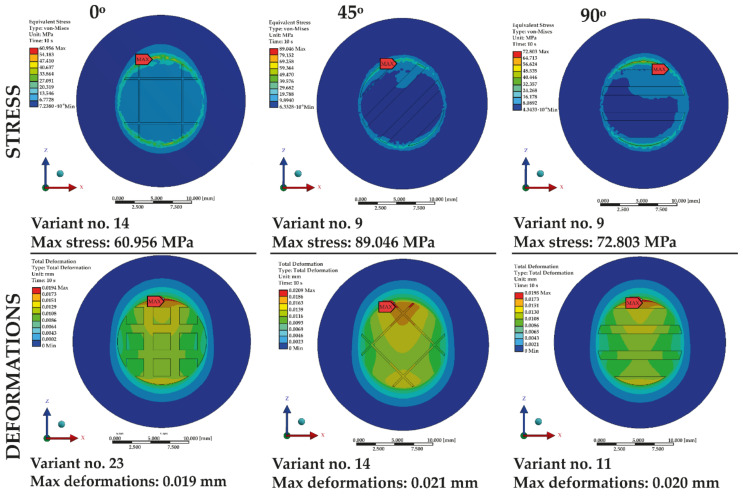
Overview of all variants which resulted in the highest values of stress and deformation.

Maps of the highest values of deformations and stresses are shown in Figure 12.

All possible configurations of grooves for the three angles of application of force were also analyzed. An overview of best values of each configuration can be found in Table 3, Table 4 and Table 5. The most favorable configurations were distinguished by examining the influence of individual parameters on the value of stresses and deformations, where both stresses and deformations were the lowest.

**Figure 13:**
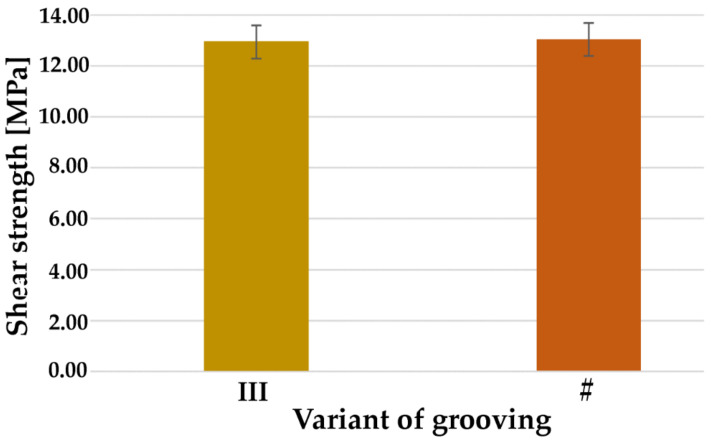
Average shear strength for sample no. 2 (marked as ‘III’) and sample no. 14 (marked as ‘#’).

The results of the shear test are shown in Figure 13. The average shear strengths in both cases were 13.0 ± 0.6 MPa.

**Figure 14:**
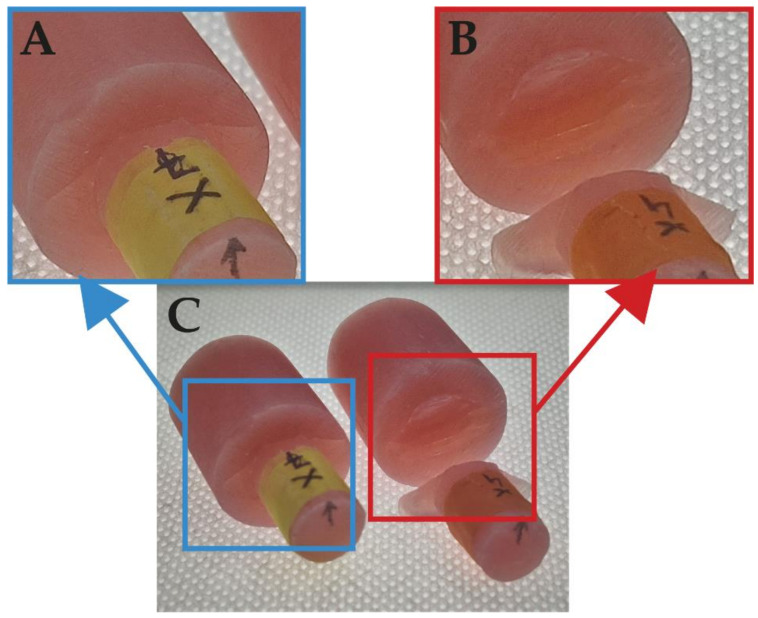
Focus on samples after the shear test: (**A**) parallel grooving (III), and (**B**) cross grooving (#); (**C**) both samples.

However, during this study, none of specimens from set III were wholly broken (Figure 14). There were only cracks inside the samples. In comparison, all samples from set # were completely damaged. These fractures were considered as mixed (partly in the sample and partly in the bonding area).

## 4. Discussion

A comparison of results at all angles shows that the worst deformations were generated in samples when the grooves were positioned at an angle of 45°. In contrast, the lowest deformation values were observed for the angle of 90°. Similar data were obtained regarding maximum stresses; in the case of 45°, they were significantly higher than in the two remaining angles.

Stress and deformation testing as a function of angle and depth (in parallel grooving) indicate that results were better when the sample was positioned at 90° and the groove depth was 0.20 mm. Regarding cross grooving, the best results were obtained when the groove depth was 0.20 mm at an angle of 0°.

Regarding stress and deformation as a function of angle and distance, an analysis of samples with cross grooving showed that the best results were generated in every combination of grooving with a distance of 5.00 mm and at an angle of 0°.

An overview of the results of stress and deformation testing as a function of angle and width indicated that with regards to parallel grooving, results were the best when the sample was positioned at an angle of 90° and the groove depth was 0.20 mm. Regarding cross grooving, the best results were produced when the width was 0.20 mm at an angle of 0°.

An analysis of the results of the simulation shows that some of the shear tests exceeded the strength of the material used, which amounts to 58.50 MPa [3,10,22]. Exceeding the strength limit of the tested samples resulted in the total break of the bond between the two components. The bond was broken in the case of the configuration marked in Table 1 as number ‘9’ at angles of 45° and 90°, as well as configuration ‘14’ at the grooving angles of 0° and 45°.

The PMMA yield limit is 51.7 MPa [3,10,22], which means that in the case of some of the examined geometries, the yield limit was exceeded, and the material was permanently plasticized. Values of stresses that exceeded the yield limit were produced in configuration ‘5’, with grooving positioned at 45° and 90° angles; in configuration ‘10’, with grooving positioned at an angle of 45°; and in configuration ‘17’, with grooving positioned at angles of 0° and 45°.

Similar results were produced by previous studies that also touched upon the issue of analyzing maximum stresses using the finite element methods [5]. The results of the tests varied in proportion to the forces applied to the models. A corresponding image of the map of the distribution of stresses and deformations in examining the impact of shearing forces was also obtained.

The highest stresses and deformations generated at the contact point of two surfaces occurred mostly at the edges of the grooves. In some cases, they appeared on a smooth connection of both cylinders.

**Figure 15:**
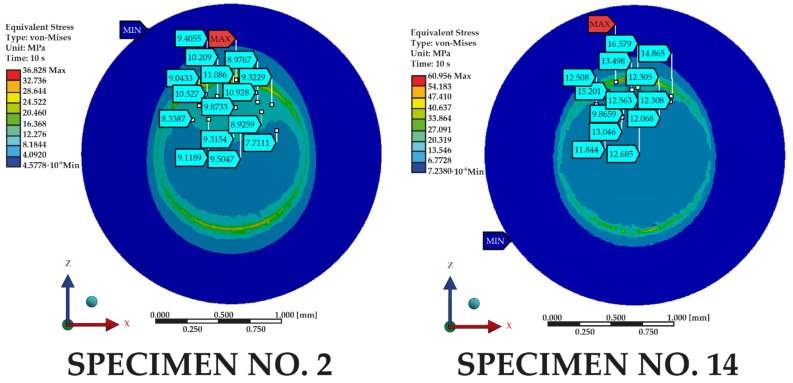
Cloud points of local stress from simulation results for specimens no. 2 and 14.

The results of the shear tests show that the appropriate shape and distribution of the grooves significantly affects the quality of the connection. Despite the achievement of the same values of MPa—the connection with III-type grooves did not break at all. There was only a crack deep into the material, but the whole structure is still connected very well. The machine automatically stopped after the force dropped, but the sample was not destroyed. The experimental tests confirmed the results of the numerical simulation analysis. Additionally, local stress measurements were made in both the numerically modeled samples, numbers 2 and 14 (Figure 15). Based on the measurement maps obtained, it is possible to notice which maximum values indicate which samples will be destroyed entirely. Otherwise, there may be only a violation of the connection of two elements, but a fragment of the sample would not be completely torn off. After a cloud points comparison using the results of the shear test, we see that specimen no. 2 obtained lover local values than no. 14, i.e., option no. 2 can pass longer loads.

## 5. Conclusions

To summarize, the finite element method enables the analysis of many grooving variants as a potential factor increasing the strength of the bond between two acrylic materials. Research shows that grooving shape affects the distribution of stresses and deformations and their values.

- The angle of 0° produces the lowest stresses in most samples, irrespective of groove dimensions.
- The lowest deformations were observed in samples with parallel grooving at an angle of 90°.
- The highest stresses were recorded when the grooving angle was 45° and the depth was 0.10 mm, indicating that increasing grooving depth reduces stresses but increases deformation.

Taking into account the groove depth of the grooves:

- In samples with parallel (III) grooves, increasing the groove depth in most cases increased the deformations but reduced stresses.
- In cross-grooved (#) variants, increased depth resulted in higher deformations but lower stresses.

An analysis of the impact of distance between grooves on stress and deformation values indicates that:

- Increasing the width of III grooves had no clear impact on results.
- The greater the distance of the grooves in the # shape, the lower the deformations and stresses.

In the case of width:

- Increasing the width results in a slight increase in deformations and stresses (III shape).
- Increasing the width has no clear impact (# shape).

**Figure 16:**
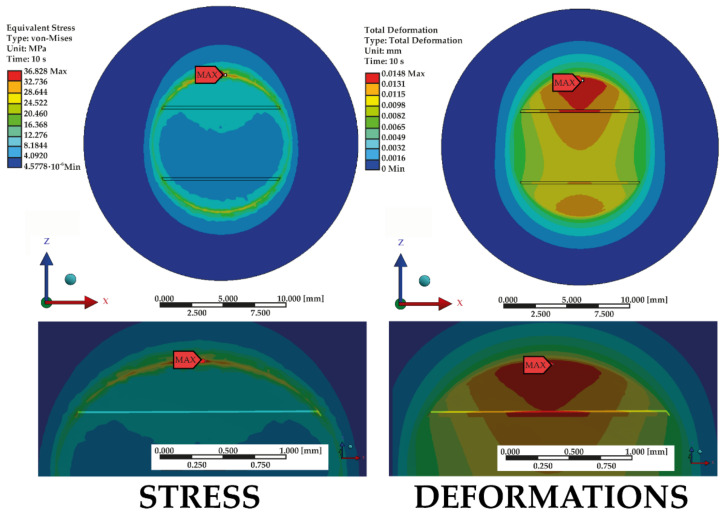
Stress and deformation maps produced in respect of variant no. 2—the optimal combination developed using numerical analysis.

**Figure 17:**
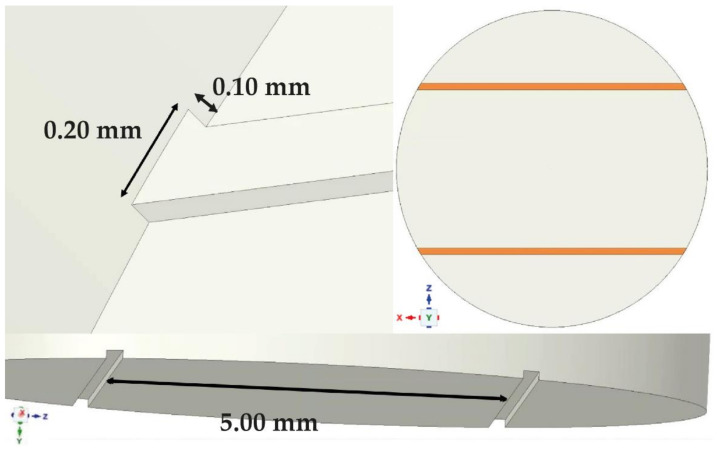
Optimal groove dimensions result in the strongest bond between acrylic materials.

This extensive analysis enables pinpointing the optimal arrangement, i.e., one characterized by both the lowest values of stress (36.828 MPa) and deformation (0.015 mm) (Figure 16), which directly impacts the improved strength of the bond. Such effects can be achieved using variant ‘2’ (Figure 17), i.e., parallel grooving with the following dimensions: width 0.20 mm, groove thickness 0.10 mm, distance between grooves 5.00 mm, shear force angle 90°. This configuration keeps stresses and deformations at a low level, even when the force is applied at an angle of 0°.

Cross grooves are a less advantageous arrangement. In these cases, low stress values did not always correspond to soft deformations. An additional advantage of parallel grooving is that it can be easily prepared in a dental lab.

Strength tests have shown that suitable grooves can extend the length of use of the structure. The entire construction with the III grooves was not destroyed, but a phased cracking occurred. The numerical simulations made it easier to select the most appropriate configurations for endurance testing. Compared to a laboratory experiment, computer simulation fares better because it is much cheaper and easier to carry out and allows us to simulate any complex problem while considering the influence of various factors, which is not always possible for a physical experiment.

This mechanical surface development method combined with an appropriate chemical substance may significantly increase the strength of the bond between two acrylic materials, which constitutes the next step in our research.

## Figures and Tables

**Table 1:** Dimensions and variants of all created geometries.

**Parallel Grooving (Marked as ‘III’)**								
**N°**	**Dimensions (mm)**		**N°**	**Dimensions (mm)**		**N°**	**Dimensions (mm)**	
1	width	0.20	5	width	0.50	9	width	1.00
	distance	2.00		distance	2.00		distance	2.00
	depth	0.10		depth	0.10		depth	0.10
2	width	0.20	6	width	0.50	10	width	1.00
	distance	5.00		distance	5.00		distance	5.00
	depth	0.10		depth	0.10		depth	0.10
3	width	0.20	7	width	0.50	11	width	1.00
	distance	2.00		distance	2.00		distance	2.00
	depth	0.20		depth	0.20		depth	0.20
4	width	0.20	8	width	0.50	12	width	1.00
	distance	5.00		distance	5.00		distance	5.00
	depth	0.20		depth	0.20		depth	0.20
**Cross Grooving (Marked as ‘#’)**								
**N°**	**Dimensions (mm)**		**N°**	**Dimensions (mm)**		**N°**	**Dimensions (mm)**	
13	width	0.20	17	width	0.50	21	width	1.00
	distance	2.00		distance	2.00		distance	2.00
	depth	0.10		depth	0.10		depth	0.10
14	width	0.20	18	width	0.50	22	width	1.00
	distance	5.00		distance	5.00		distance	5.00
	depth	0.10		depth	0.10		depth	0.10
15	width	0.20	19	width	0.50	23	width	1.00
	distance	2.00		distance	2.00		distance	2.00
	depth	0.20		depth	0.20		depth	0.20
16	width	0.20	20	width	0.50	24	width	1.00
	distance	5.00		distance	5.00		distance	5.00
	depth	0.20		depth	0.20		depth	0.20

**Table 2:** Material properties of acrylic resin used in numerical simulations.

Property	Value
Density	1.9 g/cm^3^
Poisson’s Ratio	0.44
Young’s Modulus	2 940 MPa
Yield Strength	51.7 MPa
Tensile Strength	58.5 MPa
Compressive Yield Strength	81.4 MPa

**Table 3:** The best grooving parameters as a function of depth and angle.

Grooving	Analyzed Dimensions		The Best Values Were Obtained with	
	Width (mm)	Distance (mm)	Angle (°)	Depth (mm)
Parallel	0.20	2.00	0	0.20
	0.20	5.00	90	0.10
	0.50	2.00	90	0.20
	0.50	5.00	90	0.10
	1.00	2.00	45	0.20
	1.00	5.00	90	0.20
Cross	0.20	2.00	0	0.20
	0.20	5.00	0	0.20
	0.50	2.00	0	0.20
	0.50	5.00	45	0.20
	1.00	2.00	45	0.20
	1.00	5.00	0	0.20

**Table 4:** The best grooving parameters as a function of distance and angle.

Grooving	Analyzed Dimensions		The Best Values Were Obtained with	
	Width (mm)	Depth (mm)	Angle (°)	Distance (mm)
Parallel	0.20	0.10	90	5.00
	0.20	0.20	45	5.00
	0.50	0.10	90	5.00
	0.50	0.20	90	2.00
	1.00	0.10	0	5.00
	1.00	0.20	90	5.00
Cross	0.20	0.10	0	2.00
	0.20	0.20	0	5.00
	0.50	0.10	0	5.00
	0.50	0.20	0	5.00
	1.00	0.10	0	5.00
	1.00	0.20	0	5.00

**Table 5:** The best grooving parameters as a function of width and angle.

Grooving	Analyzed Dimensions		The Best Values Were Obtained with	
	Distance (mm)	Depth (mm)	Angle (°)	Width (mm)
Parallel	2.00	0.10	45	0.20
	5.00	0.10	90	0.20
	2.00	0.20	90	0.20
	5.00	0.20	90	1.00
Cross	2.00	0.10	0	0.20
	5.00	0.10	0	0.50
	5.00	0.20	0	0.20
	5.00	0.20	0	0.20

## Data Availability

Not applicable.
